# ARV-771 Acts as an Inducer of Cell Cycle Arrest and Apoptosis to Suppress Hepatocellular Carcinoma Progression

**DOI:** 10.3389/fphar.2022.858901

**Published:** 2022-05-04

**Authors:** Yuanfei Deng, Cuifu Yu, Lushi Chen, Xin Zhang, Qiucheng Lei, Qing Liu, Gengxi Cai, Fang Liu

**Affiliations:** ^1^ Department of Pathology, The First People’s Hospital of Foshan, Foshan, China; ^2^ Guangzhou Municipal and Guangdong Provincial Key Laboratory of Protein Modification and Degradation, School of Basic Medical Sciences, Guangzhou Medical University, Guangzhou, China; ^3^ Health Management Center, The First People’s Hospital of Foshan, Foshan, China; ^4^ Department of Hepatopancreatic Surgery, The First People’s Hospital of Foshan, Foshan, China; ^5^ Department of Breast Surgery, The First People’s Hospital of Foshan, Foshan, China

**Keywords:** hepatocellular carcinoma, PROTACs, ARV-771, deubiquitinases, MAPKs

## Abstract

Hepatocellular carcinoma (HCC) is the most commonly diagnosed liver cancer with limited treatment options and extremely poor prognosis worldwide. Recently, the proteolysis targeting chimeras (PROTACs), which aim to induce proteasome-mediated degradation of interesting proteins via recruiting E3 ligases, have become the advanced tools and attractive molecules for cancer treatment. However, the anticancer effects of PROTACs in HCC remain to be clarified. Here, we evaluate the anticancer activity of ARV-771, a previously reported PROTAC compound designed for bromodomain and extra-terminal domain (BET) proteins, in HCC. We show that ARV-771 suppresses the cell viability and colony formation of HCC cells via arresting cell cycle progression and triggering apoptosis. Further investigations reveal that ARV-771 notably downregulates multiple non-proteasomal deubiquitinases which are critical to the development of cancers. Additionally, HCC cells can decrease their sensitivity to ARV-771 via activating the MEK/ERK and p38 MAPKs. ARV-771 also inhibits HCC progression *in vivo*. Moreover, we show that ARV-771 and sorafenib, a Raf inhibitor that clinically used for targeted therapy of liver cancer, can synergistically inhibit the growth of HCC cells. Overall, this study not only explores the anticancer activity of ARV-771 and its underlying mechanisms in HCC, but also deepens our understanding of deubiquitinases, MAPKs, cell cycle, and apoptosis induction in cancer therapy.

## Introduction

Liver cancer is the third leading cause of cancer-related deaths worldwide (Sung et al., 2021) that includes hepatocellular carcinoma (HCC), cholangiocellular carcinoma and secondary carcinoma of liver. HCC is the major type of liver cancer with unsatisfactory treatment options and extremely poor outcome (Zhang et al., 2021). Although sorafenib, the first-line agent that targets multiple kinases for the treatment of advanced HCC, has been clinically applied in patients for over decades, less than 10% patients respond to the therapy or even develop drug resistance(Zhu et al., 2017). In addition, the overall survival of patients with HCC can be only extended for less than 1 year by sorafenib (Forner et al., 2018). Therefore, it remains a challenge for current medicine to identify novel and more universally effective chemicals to better improve prognosis of HCC patients.

Protein stability control is mainly maintained by the balance between ubiquitination and deubiquitination. Unbalanced state of ubiquitination and deubiquitination has been proposed to involve in or even dominate the development and progression of various diseases, such as cancers (Liao et al., 2017; Liao et al., 2018; Yu et al., 2021), cardiovascular diseases (Qi et al., 2020; Rangrez et al., 2020) and neurodegenerative diseases (Zhang et al., 2020; Liu and Moussa 2021). Protein deubiquitination, a biological process in reverse to protein ubiquitination, is regulated by a class of deubiquitinases (DUBs) which contain a special deubiquitinating domain. Recently, the roles of DUBs have been highlighted in the progression of HCC. For example, the ubiquitin-specific peptidase 1 (USP1) may promote proliferation and migration of HCC via controlling the protein stability of ribosomal protein S16 (RPS16) (Liao et al., 2021b). Additionally, the USP14 can also facilitate cell proliferation and migration by maintaining HIF1-α stability in HCC (Lv et al., 2021). The USP8 is overexpressed and associated with drug resistance of HCC (Zhu et al., 2021). Deubiquitination and stabilization of YAP/TAZ controlled by USP10 facilitate the proliferation of HCC (Zhu et al., 2020). Moreover, by stabilizing different tumor drivers, OTU domain-containing protein 3 (OTUD3) (Xie et al., 2021), USP22 (Jing et al., 2021), and USP39 (Li et al., 2021) are also reported to determine the growth and progression of HCC. These reports collectively demonstrate that DUBs may be developed as important targets for HCC treatment.

Proteolysis targeting chimera (PROTAC) is a revolutionary approach for compound synthesis and a promising strategy for disease treatment. Unlike traditional protein inhibitors, PROTAC technology can target the previously undruggable proteins. The basic principle of PROTAC is to design a hybrid compound with two-tier function that can bind the proteins of interest on one side, while can also bind the E3 ligase on the other side, which ultimately induces ubiquitination and degradation of the targets (Maniaci et al., 2017; Bondeson et al., 2018). Recently, a class of small molecules based on PROTAC technology have been developed, such as ARV771(Raina et al., 2016), ARV-825(Lu et al., 2015), PROTAC SGK3 degrader-1 (Tovell et al., 2019), and UNC6852 (Potjewyd et al., 2020). ARV-771, a pan-(bromodomain and extra-terminal) BET-PROTAC, is capable of inducing degradation of bromodomain containing 2/3/4 (BRD2/3/4). ARV-771 dramatically suppresses proliferation of castration-resistant prostate cancer models via inducing Von Hippel Lindau (VHL) E3 ligase-mediated degradation of BRDs and inhibiting AR signaling (Raina et al., 2016). Subsequent study has been demonstrated that ARV-771 can also suppress proliferation of mantle cell lymphoma cells via increasing the levels of tumor suppressors, including CDKN1A/p21, HEXIM1, and NOXA (Sun et al., 2018). These novel findings illuminate that ARV-771 may exert antitumor effect in other cancers by altering some biological process related proteins or signaling pathways that are undiscovered previously.

In the current study, we show that ARV-771 can induce degradation of BET proteins, but this effect is not sufficient to suppress the growth of HCC. Nevertheless, we uncover that ARV-771 may display its lethal effect on HCC cells via inhibiting multiple DUBs. In addition, HCC cells can potentially develop resistance to ARV-771 via activating MEK/ERK and p38 kinases. Moreover, the combination of ARV-771 and sorafenib, a multi-kinase inhibitor used as the first line compound for advanced HCC treatment, synergistically inhibits proliferation of HCC cells. Collectively, this study provides a novel insight into the antitumor efficacy and molecular mechanisms of ARV-771, which may represent a promising anti-HCC strategy.

## Materiers and Methods

### Materials

ARV-771 (S8532), LY3214996 (S8534), SB230580 (S1076), SP600125 (S1460), and sorafenib (S7397) were obtained from Selleckchem (Houston, TX). Anti-CDK4 (ab108357), anti-CDK6 (ab124821), anti-Cyclin D1 (ab16663), anti-p27 (ab32034), anti-Bcl-2 (ab32124), anti-Bcl-XL (ab32370), anti-BRD2 (ab139690), anti-BRD3 (ab50818), anti-BRD4 (ab128874), anti-USP5 (ab154170), anti-USP7 (ab108931), anti-USP8 (ab228572), anti-USP9X (ab19879), anti-USP10 (ab109291), anti-USP13 (ab109624), anti-USP18 (ab168478), anti-USP39 (ab131244), anti-USP14 (ab235960), anti-UCH37 (ab133508), anti-p-ERK (ab32538), anti-ERK (ab184699), anti-p-p38 (ab4822), and anti-p38 (ab170099) were obtained from Abcam (Cambridge, MA). Anti-PARP (#9532), anti-USP1 (#8033), anti-USP15 (#66310), anti-STAMBP (#5245), anti-K48-linked ubiquitin (#12805), anti-ubiquitin (#3936), anti-SIX1 (#16960), anti-SKP2 (#2652), anti-GAPDH (#5174), anti-p-MEK (#3958), anti-MEK (#4694), anti-p-JNK (#9255), and anti-JNK (#9252) were purchased from Cell Signaling Technology (Beverly, MA).

### Cell Culture

HepG2, Hep3B, and HCCLM3 cell lines were obtained from the American Type Culture Collection (Manassas, VA, United States) and validated by short tandem repeat profiling. The above HCC cell lines were grown in RPMI 1640 medium with 10% fetal bovine serum (FBS) and cultured in the standard condition.

### Proliferation Assays

Cell viability assay using the MTS assay kit (#G3581, Promega, Madison, WI, United States) was performed to determine the short-term proliferative ability of cancer cells. EdU staining assay was performed to determine the rate of DNA duplicate in cancer cells. Clonogenic assay was conducted to determine the long-term proliferative ability of cancer cells. These assays were all conducted as a previous report (Liao et al., 2019) and done in triplicate.

### Cell Cycle Assay

Cultured HCC cells were collected and resuspended with 500 μL cold PBS and 2 ml cold 70% ethanol at 4°C for 12 h, followed by the staining with 50 μg/ml PI, 100 μg/ml RNaseA, and 0.2% Triton-X-100 mixture at 4°C for 30 min. After reaction, the HCC cells were analyzed by flowcytometry. The experiments were performed in triplicate.

### Apoptosis Assay

HCC cells exposed to ARV-771 were collected and washed with cold PBS for three times. Cells were then resuspended with 500 μL binding buffer and incubated with 5 μL annexinV-FITC and 5 μL PI in the dark for 30 min. Finally, the apoptotic cells were analyzed by flowcytometry and an inverted fluorescence microscope (AxioObsever Z1, Zeiss). The experiments were performed in triplicate.

### SiRNA Interfering Assay

HCC cells were transfected with BRD2/3/4 siRNAs or control siRNAs using lipofectamine™ RNAiMAX (Invitrogen). RPMI opti-MEM (Gibco) were applied to incubate the RNAiMAX and siRNAs for 15 min. The transfection mixture was added to HCC cells and ensured the final concentration of siRNAs was 50 nM. The sequences of siRNAs were listed below (all were provided from 5′ to 3’): si-BRD2-1: GAAGCA TGCTGCCTATGCT; si-BRD2-2: CTG​GGA​GTC​TTG​AGC​CTA​A; si-BRD3-1: GGG AGATGCTATCCAAGAA; si-BRD3-2: CAG​ATG​ACA​TAG​TGC​TAA​T; si-BRD4-1: GGA​CTA​GAA​ACT​TCC​CAA​A; si-BRD4-2: TGC​TCA​GAG​TGG​TGC​TCA​A.

### Quantitative Proteomics Analysis

HepG2 cells were treated with ARV-771 0.5 μM or vehicle for 24 h. In this assay, three biological repeats were performed. A Pierce BCA Protein Assay Kit was used to determine the protein concentration. For quality control, 20 μg proteins in each sample were separated with 12% SDS-PAGE, and followed by Coomassie staining. And then, 150 μg proteins in each sample were dissolved and denatured with 200 ul of 8M urea in Nanosep Centrifugal Devices (PALL). After centrifugation, proteins were reduced with 10 mM DTT for 2 h at 56°C. Subsequently, the samples were incubated in 5 mM iodoacetamide for 30 min in the dark to block reduced cysteine residues followed by centrifugation. The digests were collected by centrifugation and labelled with iTRAQ reagent (AB Sciex, United States). After pooling, the samples were evaporated by vacuum concentration to remove excess water, TEAB, and isopropanol, followed by the chromatographic resolution and LC-MS/MS analysis. KEGG (Kyoto Encyclopedia of Genes and Genomes, https://www.kegg.jp) database was used for gene enrichment and pathway analysis.

### Immunoblot Assay

Immunoblot assay was performed as previously reported (Liao et al., 2021a). In short, pan proteins were extracted from HCC cells using RIPA buffer supplemented with phosphatase inhibitor cocktail on the ice. Cell lysates were then sonicated for three times (10 s/time) to break the DNA. Protein determination was performed using BCA assay. After determination and denaturation, 20 μg proteins were loaded onto and disassociated by 12% SDS–PAGE gels, followed by the transference to polyvinylidene difluoride membranes and blockade with non-fat 5% milk for 1 h. Then, the membranes were washed with PBS-T for three times and incubated with diverse primary antibodies at 4°C overnight. The working concentration of primary antibodies were 1:1,000. After the reaction and wash with PBS-T, all membranes were incubated with horseradish peroxidase (HRP)-conjugated secondary antibodies for 1 h. Finally, the membranes were reacted to the ECL detection reagents and exposed to X-ray films (Kodak, Japan). Each membrane was stripped less than thrice.

### Animal Study

5–6 weeks old nude mice were purchased from Vital River Laboratory Animal Technology Co., Ltd. (Beijing, China). Animal study was performed at the Laboratory Animal Center of Guangzhou Medical University in compliance with the principles of animal ethical treatment. Firstly, HepG2 cells were digested, washed, and collected in cold PBS. Then, 2 × 10^6^ cells in 100 μL PBS were subcutaneously inoculated on each nude mouse within 30 min. The nude mice successfully bearing transplanted tumors were randomly divided into two groups (8 mice/group), and treated with ARV-771 (20 mg/kg/day, i.h.) or control solvent every other day. These mice were sacrificed by humanistic vertebrate dislocation after inhalation of CO_2_, post the treatment of ARV-771 for 25 days. Tumor volume and body weight of the mice were measured every 5 days. The tumor weight was measured immediately by an electronic balance after the xenografts were extracted from the mice.

### H&E Staining and Immunohistochemistry

The liver, kidney, and xenograft were taken from the nude mice treated with ARV-771 or control solvent after they were sacrificed. Liver, kidney and tumor tissues were cut in an appropriate size and fixed with paraformaldehyde for 3 days. Then, the tissues were dehydrated, permeabilized, and embedded with paraffin according to standard techniques. The embedded liver and kidney tissues were sectioned at 4 μm/slide, and stained with hematoxylin and eosin after deparaffinage to observe the morphological alterations. The immunohistochemistry assay was carried out using a MaxVision Kit (Maixin Biol) in the embedded and sectioned xenograft tissues according to the manufacturer’s instruction. Primary antibodies in this study include anti-Cyclin D1, anti-cleaved Caspase 3, and anti-Ki67. The ImageJ software was utilized to analyze protein expression in tissues.

### Combination Index

The combination index (CI) was used to evaluate the interaction between two chemicals and calculated by the Chou-Talalay equation (Chou and Talalay 1984). CI value less than 1 indicates synergistic effect; CI value equal to 1 indicates an additive effect; and CI value more than 1 indicates antagonistic effect.

### Data Analysis

The statistical significance of data (presented as mean ± SD where applicable) was evaluated by student’s t-tests or one-way ANOVA. SPSS 16.0 and GraphPad Prism 7.0 were applied for data analysis. A two-sided *p* value of <0.05 was considered as statistically significant.

## Results

### ARV-771 Inhibits the Growth of Hepatocellular Carcinoma Cells

**FIGURE 1:**
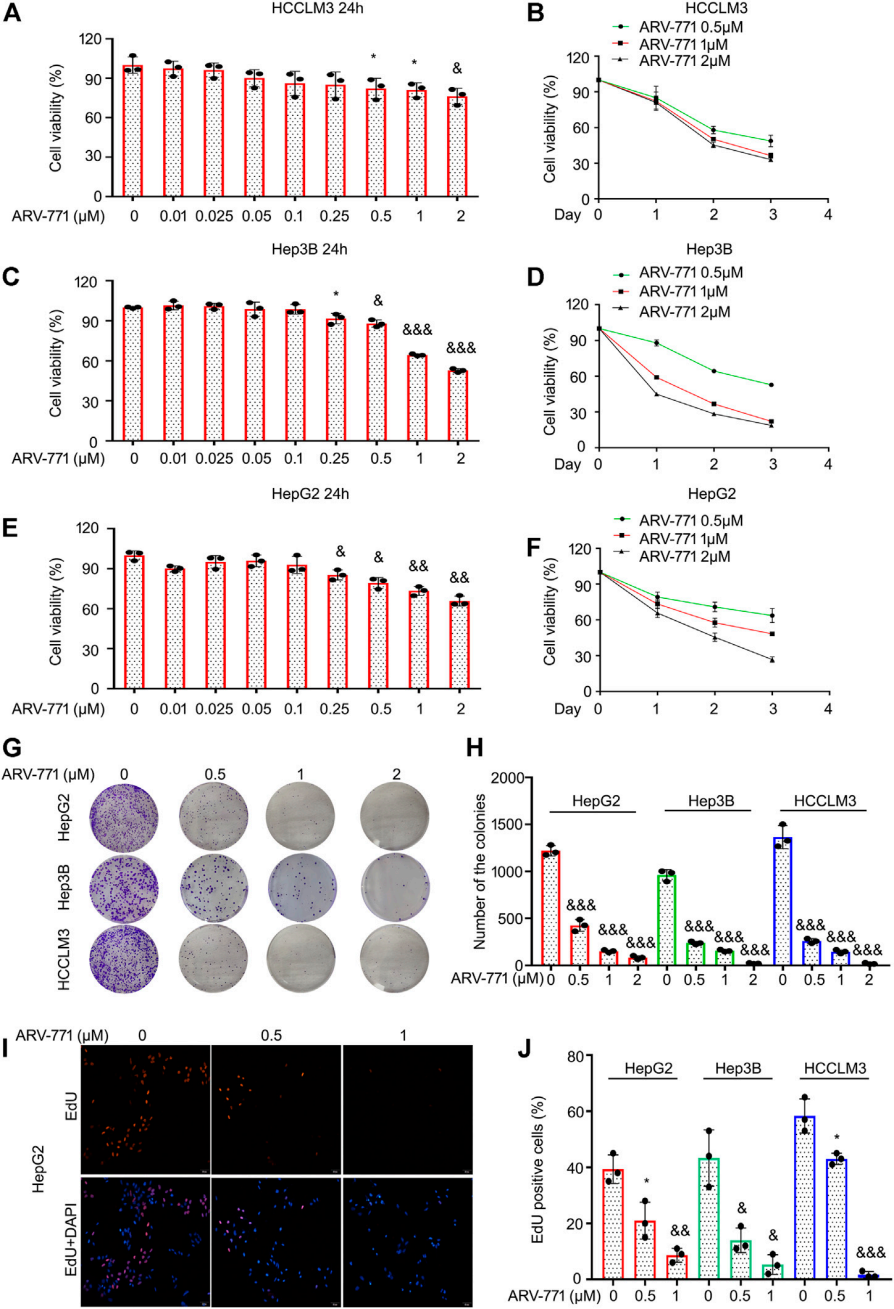
ARV-771 blocks proliferation in HCC cells. **(A)–(F)** Cell viability assays were conducted in HCCLM3, Hep3B, and HepG2 cells treated with ARV-771. **(G)** Clonogenic assays were conducted in the indicated HCC cells exposed to ARV-771 for 2 weeks. **(H)** Quantification of colony numbers were shown. **(I)** EdU staining assays were performed in the indicated HCC cells for 24 h. Representative images were shown. **(J)** Quantification of EdU positive cells were shown. ^*^ *p* < 0.05, ^&^ *p* < 0.01, ^&&^ *p* < 0.001.

The anticancer activities of PROTACs, the advanced biological tools which aim to induce degradation of targeted proteins via recruiting E3 ligases, in HCC remain elusive. To explore the antitumor effects of ARV-771 in HCC cells, cell viability assays were firstly performed in various HCC cell lines, including Hep3B and HepG2, exposed to ARV-771 for 24, 48, and 72 h. The results showed that ARV-771 dose-dependently inhibited cancer cell viability when its concentration reached at 0.25 μM in Hep3B and HepG2 cells, while reached at 0.5 μM in HCCLM3 cells. Meanwhile, ARV-771 time-dependently suppressed cell viability in HCC cells (Figures 1A–F). Next, clonogenic assay assays were performed in Hep3B, HepG2, and HCCLM3 cells for 2 weeks to determine the long-term antitumor effects of ARV-771 on HCC. We showed that ARV-771 dose-dependently decreased numbers of the colonies in above cell lines (Figures 1G,H). Furthermore, EdU staining assays were conducted in the above HCC cells to determine the rate of DNA replicate post the treatment of ARV-771. We found that the EdU positive cells were decreased by the exposure of ARV-771, indicating the rate of DNA replicate were declined in HCC cells (Figures 1I,J). Together, our findings demonstrate that ARV-771 exerts favorable antitumor activity in HCC.

### ARV-771 Blocks Cell Cycle Progression of Hepatocellular Carcinoma Cells

**FIGURE 2:**
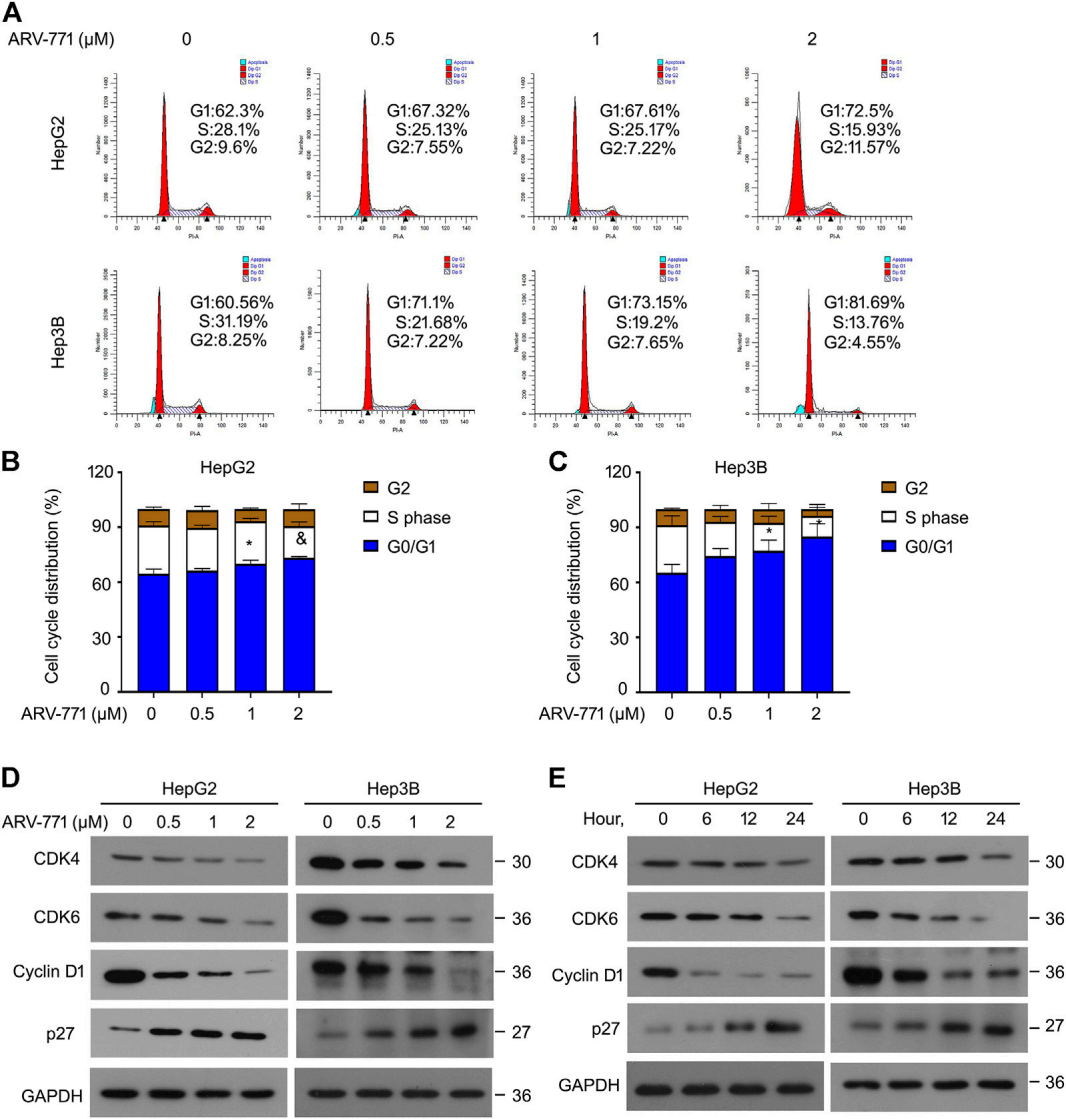
ARV-771 postpones cell cycle transition in HCC cells. **(A)** Cell cycle assays were conducted in the indicated HCC cells exposed to ARV-771 for 24 h **(B)–(C)** Quantitative cell cycle distributions in the indicated HCC cells. ^*^ *p* < 0.05, ^&^ *p* < 0.01. **(D)** Immunoblot assays for the indicated cell cycle players were performed in HepG2 and Hep3B cells treated with ARV-771 (0.5, 1, 2 μM) for 24 h. **(E)** Immunoblot assays for the indicated cell cycle players were performed in the indicated HCC cells exposed to ARV-771 (2 μM) for 6, 12, and 24 h.

Highly active cell cycle progression, an abnormal biological progress to meet the needs of rapid proliferation, is a common characteristic of cancer. To address whether ARV-771 could affect cell cycle progression of HCC cells, cell cycle assays using propidium iodide (PI) staining and flowcytometry analysis were firstly performed in HCC cells. Our data showed that the cell cycle distribution at G0/G1 phase was more in ARV-771-treated group than that in the vehicle group, whereas the cell cycle distribution at S phase was less in ARV-771-treated group than that in the vehicle group in both HepG2 and Hep3B cells (Figures 2A–C). Interestingly, we found that ARV77-1 did not alter the distribution of G0/G1 phase, but mainly induced G2/M phase arrest in HCCLM3 cells (Supplementary Figures S1A), suggesting that ARV-771 exerts different effects in HCCLM3 cells. To further explore whether ARV-771 could alter the expression of the related genes that contribute to the transition from G0/G1 to S phase, western blot assays to CDK4/6, Cyclin D1 (cell cycle drivers), and p27 (cell cycle inhibitor) were performed in HepG2 and Hep3B cells post ARV-771 exposure. The results showed that ARV-771 reduced the protein levels of CDK4/6 and Cyclin D1, while increased the protein level of p27 in a dose- and time-dependent manner (Figures 2D,E). Together, our data demonstrate that ARV-771 may suppress growth of HCC cells via leading to cell cycle arrest.

### ARV-771 Induces Apoptosis of Hepatocellular Carcinoma Cells

**FIGURE 3:**
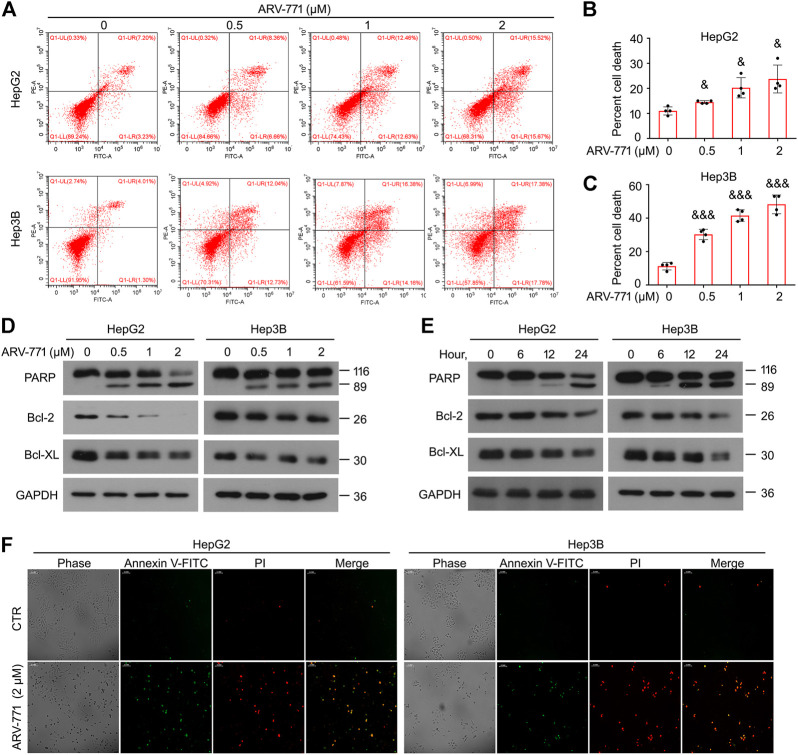
ARV-771 triggers apoptosis in HCC cells. **(A)** Apoptosis assays were performed in HepG2 and Hep3B cells post the treatment of ARV-771 for 24 h **(B)–(C)** Quantitative cell death was shown. ^&^ *p* < 0.01, ^&&&^ *p* < 0.0001. **(D)** Immunoblot assays for the indicated apoptosis-associated proteins were performed in HepG2 and Hep3B cells treated with ARV-771 (0.5, 1, 2 μM) for 24 h. **(E)** Immunoblot assays for the indicated apoptosis-associated proteins were performed in HepG2 and Hep3B cells treated with ARV-771 (2 μM) for 6, 12, and 24 h. **(F)** Annexin V-FITC/PI staining assays were performed in HepG2 and Hep3B cells treated with ARV-771 for 24 h.

Resistant to apoptosis is another common characteristic which is critical to cancer development and progression. To further determine whether ARV-771 could induce apoptosis in HCC, the canonical apoptosis assays using annexin V-FITC/PI staining combined with flowcytometry analysis were performed in HepG2 and Hep3B cells post ARV-771 exposure. The results showed that ARV-771 significantly triggered apoptosis in both HCC cell lines (Figures 3A–C). However, we showed that ARV-771 failed to trigger apoptosis in HCCLM3 cells (Supplementary Figure S1B), indicating that ARV-771 induced a selective apoptosis in HCC cells in some unknown mechanism. Next, we determined the expression of multiple apoptosis-associated proteins, including PARP (a well-defined biomarker for apoptosis), Bcl-2, and Bcl-XL (two mitochondria-associated apoptosis inhibitors) using western blot assays. We found that ARV-771 not only remarkably induced PARP cleavage, but also reduced the expression of Bcl-2 and Bcl-XL in both HepG2 and Hep3B cell lines (Figures 3D,E). Moreover, our morphological data showed that both HCC cell lines notably displayed typical characteristic of apoptosis, including cell shrinkage, phosphatidyl evagination (annexin V-FITC positive, green), and membrane fracture (PI positive, red) post ARV-771 treatment (Figure 3F). Taken together, apoptosis induction is an important path to ARV-771 mediated proliferation suppression in HCC.

### ARV-771 Decreases the Expression of Various DUBs

**FIGURE 4:**
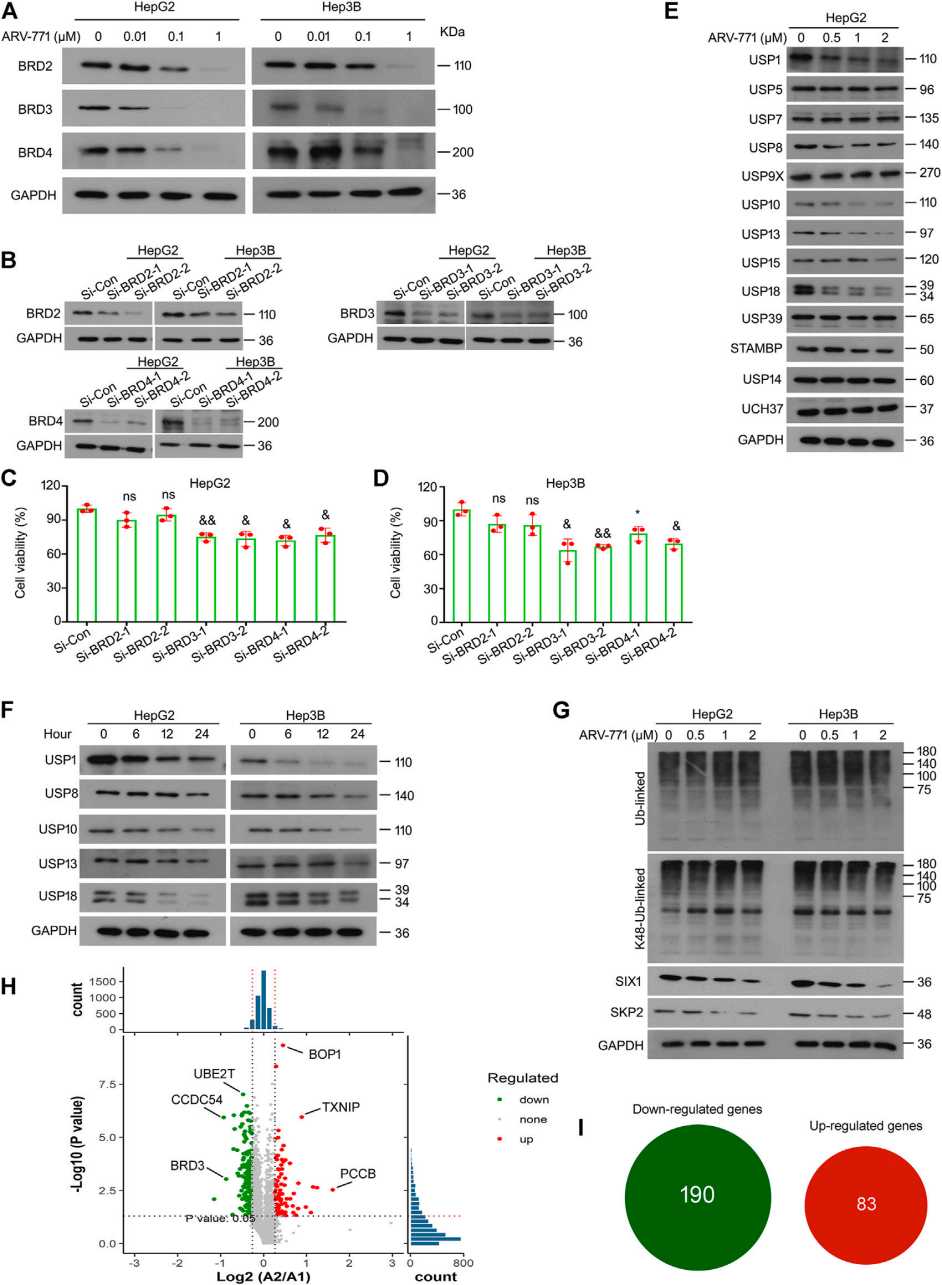
ARV-771 decreases expression of multiple deubiquitinases in HCC cells. **(A)** Immunoblot assays for BRDs were performed in HepG2 and Hep3B cells treated with ARV-771 (0.01, 0.1, 1 μM) for 24 h **(B)** HepG2 and Hep3B cells were transfected with BRDs siRNA or control siRNA for 48 h. Immunoblot assays for BRD2/3/4 were performed. **(C)–(D)** Cell viability assays were performed in HCC cells transfected with BRDs siRNA or control siRNA for 48 h ^*^ *p* < 0.05, ^&^ *p* < 0.01, ^&&^ *p* < 0.001. **(E)** Immunoblot assays for the indicated deubiquitinases were performed in HepG2 cells treated with ARV-771 (0.5, 1, 2 μM) for 24 h. **(F)** Immunoblot assays for the indicated deubiquitinases were performed in HepG2 and Hep3B cells treated with ARV-771 (2 μM) for 6, 12, and 24 h. **(G)** Immunoblot assays for pan-ubiquitinated and Lys48-linked ubiquitinated proteins, SIX1, and SKP2 were performed in HepG2 and Hep3B cells treated with ARV-771 (0.5, 1, 2 μM) for 24 h. **(H)** Quantitative volcano plot of the biological mass spectrometry performed in HepG2 cells treated with ARV-771 0.5 μM or vehicle for 24 h. A2 represents ARV-771 treatment, while A1 represents the control group. **(I)** Numbers of Up/Down -regulated genes by the treatment of ARV-771.

ARV-771, a small compound based on the PROTAC technology, is designed for the degradation of BET proteins. ARV-771 restrains the proliferation of CRPC cells by triggering protein ubiquitination and degradation of BRD2, BRD3, and BRD4. In order to determine whether ARV-771 could induce the degradation of BRD2/3/4 in HCC cells, western blot assays were performed in HepG2 and Hep3B cells post the exposure of various concentrations of ARV-771. The data showed that ARV-771 markedly decreased protein levels of BRD2, BRD3, and BRD4 when the concentration reached at 0.1 μM in HCC cells (Figure 4A). Interestingly, as we have mentioned in Figure 1, ARV-771 suppressed proliferation when its concentration reached at 0.25 μM. Next, we wondered whether BRD2, BRD3, and BRD4 are critical to HCC development. We analyzed the cell viability of HCC cells after the knockdown of BRD2, BRD3, or BRD4 using siRNA interfering assay. We found that knockdown of BRD3 and BRD4, but not BRD2, significantly decreased the proliferative ability of HCC cells (Figures 4B–D). In addition, we wondered whether knockdown of BRD3/4 could induce cell cycle arrest or apoptosis in HCC cells. However, we found that knockdown of BRD3/4 did not significantly alter cell cycle or apoptosis of HepG2 and Hep3B cells (Supplementary Figures S2A, B). Meanwhile, knockdown of BRD3/4 did not notably alter the expression of CDK4/6, Cyclin D1, and Bcl-2 (Supplementary Figure S2C), indicating that BRD3/4 promotes proliferation of HCC cells independent of cell cycle and apoptosis escape, and that ARV-771 induced cell cycle arrest and apoptosis independent of BRD3/4.

DUBs, a superfamily that functions to reverse the ubiquitination of targeted proteins, have been recently emerged as novel targets for cancer therapy due to its overexpression or high activation in various cancers. To determine whether ARV-771 could alter the expression of DUBs, western blot assays were performed to detect the expression of numerous DUBs in HepG2 cells post ARV-771 exposure. Our data showed that protein levels of non-proteasomal DUBs, including USP1, USP8, USP10, USP13, and USP18, but not other DUBs listed in the figure, was negatively fluctuated with the dose increment of ARV-771 (Figure 4E). Further investigations showed that the expression of USP1, USP8, USP10, USP13, and USP18 was negatively fluctuated with the exposure time of ARV-771 (Figure 4F). Given that ARV-771 decreased the expression of the above DUBs, we next wondered whether the overall level of protein ubiquitination could be altered by ARV-771. Our western blot assays showed that either the overall ubiquitination or the K48-linked ubiquitination level had not been altered post ARV-771 exposure in HepG2 and Hep3B cells. Instead, ARV-771 decreased the expression of SIX1 (a substrate of USP1) and SKP2 (a substrate of USP10 and USP13) (Figure 4G). These findings collectively demonstrate that ARV-771 may suppress growth of HCC via reducing the expression of USP1, USP8, USP10, USP13, and USP18 without altering the overall balance of ubiquitination control or leading to proteasome inhibition.

As we reported above, ARV-771 may exert potential off-target effects in HCC. We further performed the high-throughput biomass spectrometry analysis in HepG2 cells treated with ARV-771 or vehicle control. We found that 190 genes were downregulated (Such as BRD3, CCDC54, and UBE2T), while 83 genes were upregulated (Such as TXNIP, BOP1, and PCCB) post the treatment of ARV-771 (Figures 4H,I). KEGG analysis further showed that ARV-771 may dominantly alter three cellular processes: including cell cycle, apoptosis, p53 signaling pathway (Supplementary Figure S3). These findings are consistent with our results, and further support our hypothesis that there are limited off-target effects of ARV-771.

### Hepatocellular Carcinoma Cells Potentially Decrease Their Responsiveness to ARV-771 by Activating Mitogen-Activated Protein Kinases

**FIGURE 5:**
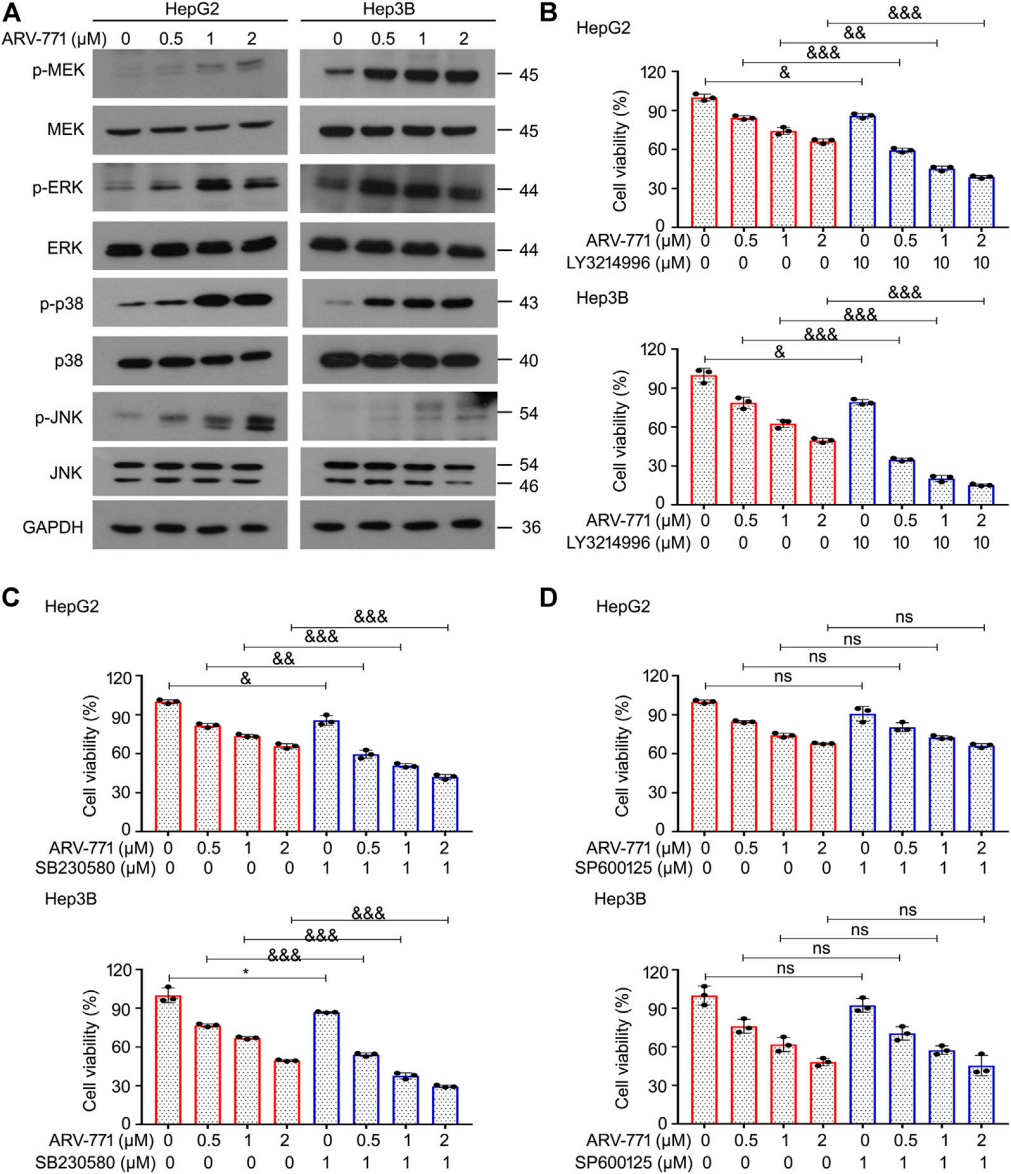
MAPK signaling protects HCC cells from the ARV-771-induced damage. **(A)** Immunoblot analyses for the indicated kinases were conducted in HCC cells post the exposure of ARV-771 (0.5, 1, 2 μM) for 24 h **(B)–(D)** Cell viability assays were conducted in HepG2 and Hep3B cells treated with ARV-771 in the presence or absence of LY3214996, SB230580, and SP600125 for 24 h ^*^ *p* < 0.05, ^&^ *p* < 0.01, ^&&^ *p* < 0.001, ^&&&^ *p* < 0.0001.

Mitogen-activated protein kinases (MAPKs) play critical roles in cell proliferation via regulating cell cycle, apoptosis, and other biological processes. Activation of the MAPKs may drive the occurrence, development, and drug resistance of hepatocellular carcinoma. Currently, numerous outstanding chemicals have been reported to exert their antitumor activity through altering the activity of MAPKs. In addition, many of which have been investigated in diverse clinical trials, e.g. VX-702 (Phase 2), PH-797804 (Phase 2), Mirdametinib (Phase 2), AZD8330 (Phase 1), Binimetinib (Phase 3). To further determine whether ARV-771 could alter the expression or function of MAPKs, western blot assays were performed to examine the expression of p-MEK, MEK, p-ERK, ERK, p-p38, p38, p-JNK, and JNK in HepG2 and Hep3B cells post ARV-771 exposure. The results showed that the expression of p-MEK, p-ERK, p-p38, and p-JNK were all upregulated by ARV-771 (Figure 5A), suggesting that ARV-771 potentially activate MAPKs signaling in HCC. To further explore the roles of these MAPKs in ARV-771-induced proliferation suppression, cell viability assays were performed in HCC cells post ARV-771 exposure with or without the addition of LY3214996 (an ERK inhibitor), SB230580 (a p38 inhibitor), or SP600125 (a JNK inhibitor). We found that LY3214996 and SB230580, but not SP600125, can increase the responsiveness of ARV-771 in both HepG2 and Hep3B cell lines (Figures 5B–D). These findings collectively illustrate that the MEK/ERK and p38 signals were activated to decrease the responsiveness of HCC cells to ARV-771.

### ARV-771 Suppresses the Growth of Hepatocellular Carcinoma Xenografts

**FIGURE 6:**
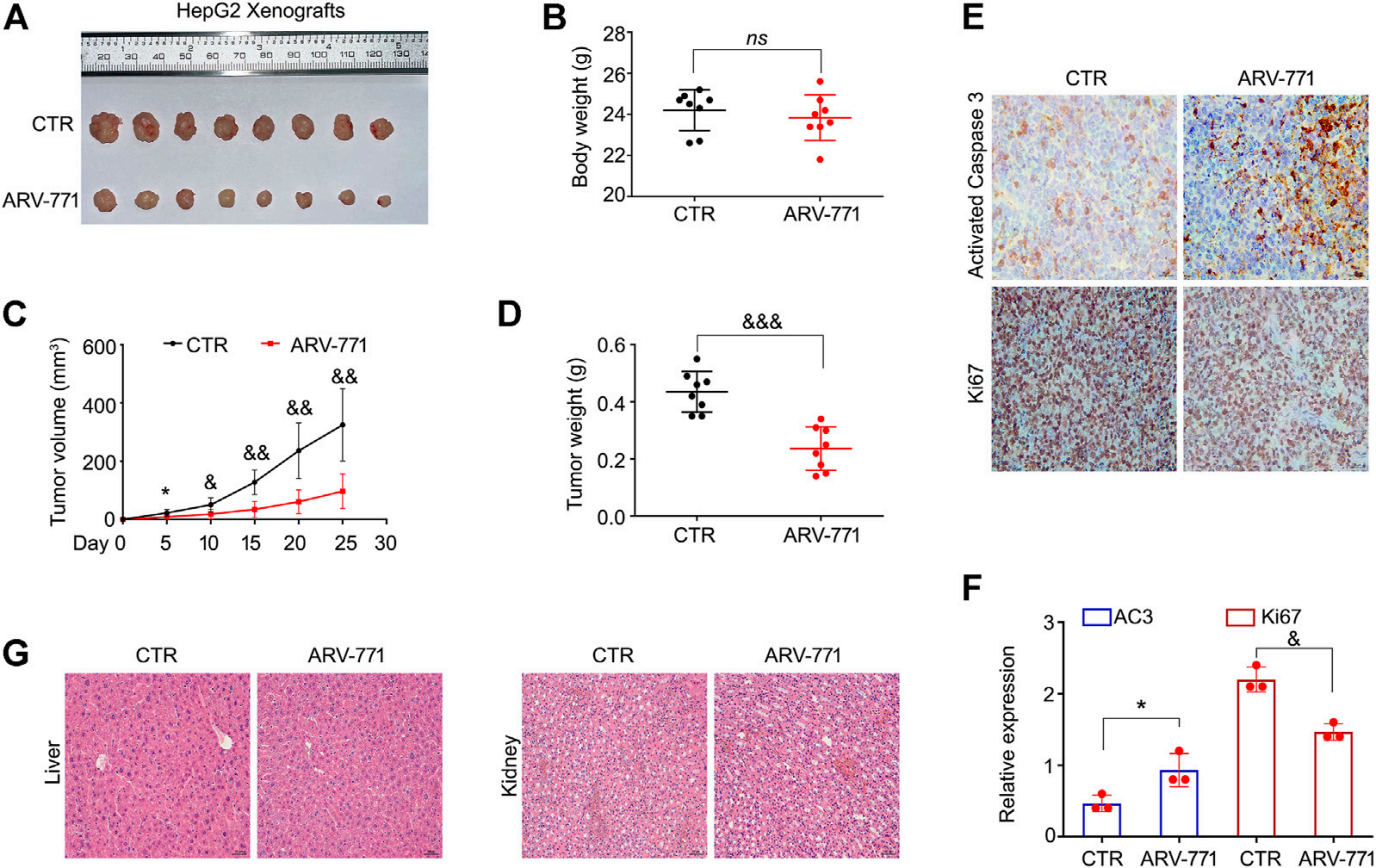
ARV-771 inhibits HCC progression in nude mouse models. **(A)** HepG2 xenograft models were established and treated with ARV-771 or vehicle for 25 days. The xenograft image was shown. **(B)** Body weight of nude mice was shown. **(C)–(D)** Tumor volumes and tumor weight were quantified. **(E)** Immunohistochemistry assays were performed in xenograft tissues. Representative images of Ki67 and activated Caspase three were shown. **(F)** Quantification of the immunohistochemistry images was shown. AC3, activated Caspase 3. ^*^ *p* < 0.05, ^&^ *p* < 0.01**. (G)** H&E staining assays were performed in liver and kidney tissues of nude mice. Representative images were shown.

To determine the anti-HCC effects of ARV-771 *in vivo*, HepG2 cells were injected in nude mice to establish HCC xenograft and treated with ARV-771 or control solvent for 25 days. We found that the tumor became smaller post the treatment of ARV-771 (Figure 6A). Meanwhile, the body weight of mice was not altered by ARV-771 (Figure 6B). Additionally, we showed that the tumor volume and tumor weight was remarkably reduced by ARV-771 (Figures 6C,D). The immunochemistry assay showed that the expression of Ki67 was reduced, while the expression of cleaved Caspase three was increased by ARV-771 in the xenograft tissues (Figures 6E,F). Moreover, the H&E staining analysis showed that ARV-771 did not obviously cause the cell damage in the liver and kidney of nude mice (Figure 6G). Taken together, our findings demonstrate that ARV-771 displays a well anti-HCC activity without severe side effects.

### ARV-771 and Sorafenib Synergistically Suppresses the Proliferation of Hepatocellular Carcinoma Cells

**FIGURE 7:**
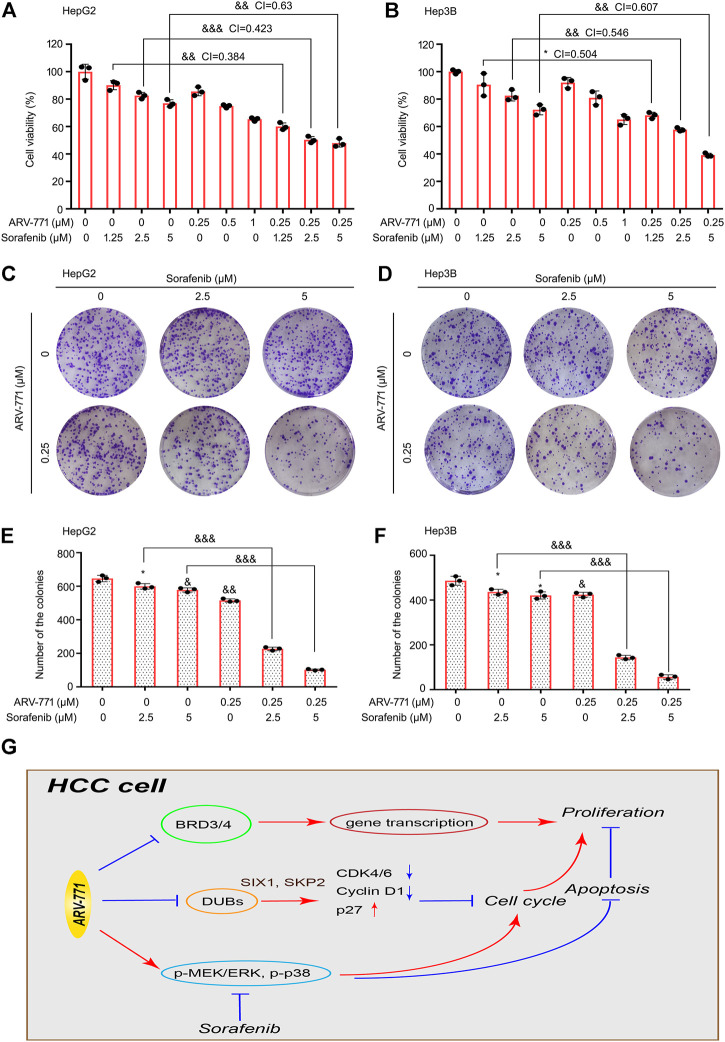
ARV-771 elevates the effectiveness of sorafenib to HCC cells. **(A)–(B)** Cell viability assays were conducted in HepG2 and Hep3B cells exposed to ARV-771 with or without sorafenib for 24 h. CI, combination index (CI value less than 1 means the synergistic effect between two drugs). ^*^ *p* < 0.05, ^&&^ *p* < 0.001, ^&&&^ *p* < 0.0001. **(C)–(D)** Clonogenic assays were conducted in HepG2 and Hep3B cells exposed to ARV-771 with or without sorafenib for 2 weeks. **(E)–(F)** Quantification of colony numbers were shown. **(G)** A molecular model for ARV-771 blocks proliferation of HCC.

Although the Raf inhibitor sorafenib is clinically used as a targeted drug for the treatment of advanced liver cancer, few patients are sensitive to this therapy. Therefore, developing a strategy to enhance the responsiveness of HCC cells to sorafenib is urgent. To further determine whether ARV-771 could increase the responsiveness of HCC cells to sorafenib, cell viability assays were performed in HepG2 and Hep3B cells post the exposure of sorafenib (1.25, 2.5, 5 μM) with or without the addition of ARV-771 (0.25, 0.5, 1 μM) for 24 h. We found that ARV-771 and sorafenib synergistically suppressed the cell viability of HCC cells (all CI values were less than 1) (Figures 7A, B). Next, colony formation assays were performed in HCC cells post the exposure of sorafenib with or without the addition of ARV-771. Consistent with the previous findings, we showed that ARV-771 and sorafenib synergistically inhibited the colony formation in both HCC cell lines (Figures 7C–F). These results collectively indicate that ARV-771 can elevate the effectiveness of sorafenib to HCC cells, which further provides a new strategy to expand the application range of sorafenib for HCC treatment in future.

## Disccussion

Identifying more effective anticancer approaches for HCC is challenging and urgent. In this study, we uncover that ARV-771 not only inhibits growth of HCC cells via inducing cell cycle blockade, apoptosis, and downregulation of multiple DUBs, but also aggravates the proliferation suppression effect induced by sorafenib in HCC cells.

Cell cycle disruption caused by highly expressed or active cyclin-dependent kinases (CDKs) and apoptosis escape caused by the unbalanced expression of pro-apoptotic and antiapoptotic proteins are observed in various types of cancer (Gerl and Vaux 2005; Lapenna and Giordano 2009; Delbridge and Strasser 2015; Mohammad et al., 2015). A pioneering study discovered that ARV-771 is a novel VHL E3 ligase-based BET PROTAC which can force degradation of BRD2, BRD3, and BRD4. The downregulation of these molecules further notably leads to the suppression of androgen receptor (AR) signaling and apoptosis in CRPC models (Raina et al., 2016). Similar to these results, our findings showed that ARV-771 can also induce apoptosis via reducing the expression of Bcl-2 and Bcl-XL, two well-characterized antiapoptotic proteins that belong to Bcl-2 family in the mitochondria. But unlike their findings, we showed that ARV-771 may affect cell cycle process via altering protein levels of CDK4/6, Cyclin D1, and p27 in HCC. Besides, other group has also been demonstrated that ARV-771 elevates the levels of cell cycle regulator (p21) and mitochondria associated apoptosis regulator (NOXA) in the mantle cell lymphoma cells (Sun et al., 2018). These findings demonstrate that ARV-771 exerts additional anticancer effects (off-target effects) via affecting the expressions of more cell cycle and apoptosis -related proteins beyond BRDs and AR in different cancers. Our biomass spectrometry analysis showed that there are limited off-target effects of ARV-771 which contributes to cell cycle arrest and apoptosis in HCC cells.

Recently, increasing DUBs were identified as tumor promoters via deubiquitinating and stabilizing certain oncoproteins in cancers, and were proposed as novel therapeutic targets. In this study, we showed that ARV-771 can downregulate multiple DUBs in HCC, including USP1, USP8, USP10, USP13, and USP18, which have been implicated in the tumorigenesis and progression by regulating certain substrates (Cai et al., 2017; Gao et al., 2020; Zhu et al., 2020; Liao et al., 2021b; Zhu et al., 2021). Therefore, we hypothesized that the reduction of USP1, USP8, USP10, USP13, and USP18 may all involve in the ARV-771-induced growth inhibition in HCC. Meanwhile, our findings also support inhibition of DUBs as a promising strategy for HCC treatment. Moreover, we showed that the cell cycle-related downstream substrates of USP1, USP10, and USP13, including SIX1 and SKP2, were downregulated by ARV-771. SIX1 is a nuclear transcriptional factor that can induce the expression of Cyclin D1 to promote cell cycle transition. SKP2 is a E3 ligase that can trigger the degradation of p27 to drive cell cycle from G0/G1 to S transition. These findings further demonstrate that inhibition of DUBs is involved in cell cycle arrest resulted from ARV-771 treatment in HCC.

MAPKs are highly conserved during the evolution of species, which is critical to cell proliferation and apoptosis. The roles of MAPKs are complicated in the tumorigenesis. In the current study, we discovered that HCC cells can potentially activate MAPKs, including p-MEK, p-ERK, and p-p38, to protect themselves against the ARV-771-induced damage, indicating that the activation of MEK/ERK and p38 signaling pathways exert a protective role in HCC cells under some hijacked conditions. Therefore, these findings were consistent with the canonical roles of MEK/ERK and p38 signaling pathways in driving cancer development and drug resistance (Bradham and McClay 2006; Samatar and Poulikakos 2014).

Although sorafenib has become the first-line targeted agent for advanced HCC, its efficacy on HCC patients is extremely limited. This cruel reality prompted us to explore a promising compound to enhance the efficacy of sorafenib. As a multi-kinase inhibitor, sorafenib mainly inactivates the Raf-1 and B-Raf kinases (Wilhelm et al., 2004), and their direct or indirect downstream effectors, including the MEK/ERK and p38 MAPKs (Wilhelm et al., 2004; Zhang et al., 2018). Fortunately, combined with our previous findings that the activation of both MEK/ERK and p38 signaling pathways restrained the efficacy of ARV-771, we spontaneously explored the effect of sorafenib in combination with ARV-771 in HCC cells. We showed that ARV-771 and sorafenib synergistically suppressed the growth of HCC, supporting our novel notion that taking the complementary advantages between two agents as a drug combination strategy for cancer therapy.

In summary, our findings strongly demonstrate the novel activities of ARV-771 in HCC (Figure 7G), which may further broaden the application range of the PAOTAC-based molecules for cancer treatment and provide a promising therapeutic strategy in HCC.

## Data Availability

The original contributions presented in the study are included in the article /Supplementary Material, further inquiries can be directed to the corresponding authors.
