# Supplementary material for: ARV-771 Acts as an Inducer of Cell Cycle Arrest and Apoptosis to Suppress Hepatocellular Carcinoma Progression
Source: Front Pharmacol. 2022 May 4;13:858901. doi: 10.3389/fphar.2022.858901 (PMC9114478; doi:10.3389/fphar.2022.858901)
Supplement: Supplementary file 1 [file DataSheet1.docx]

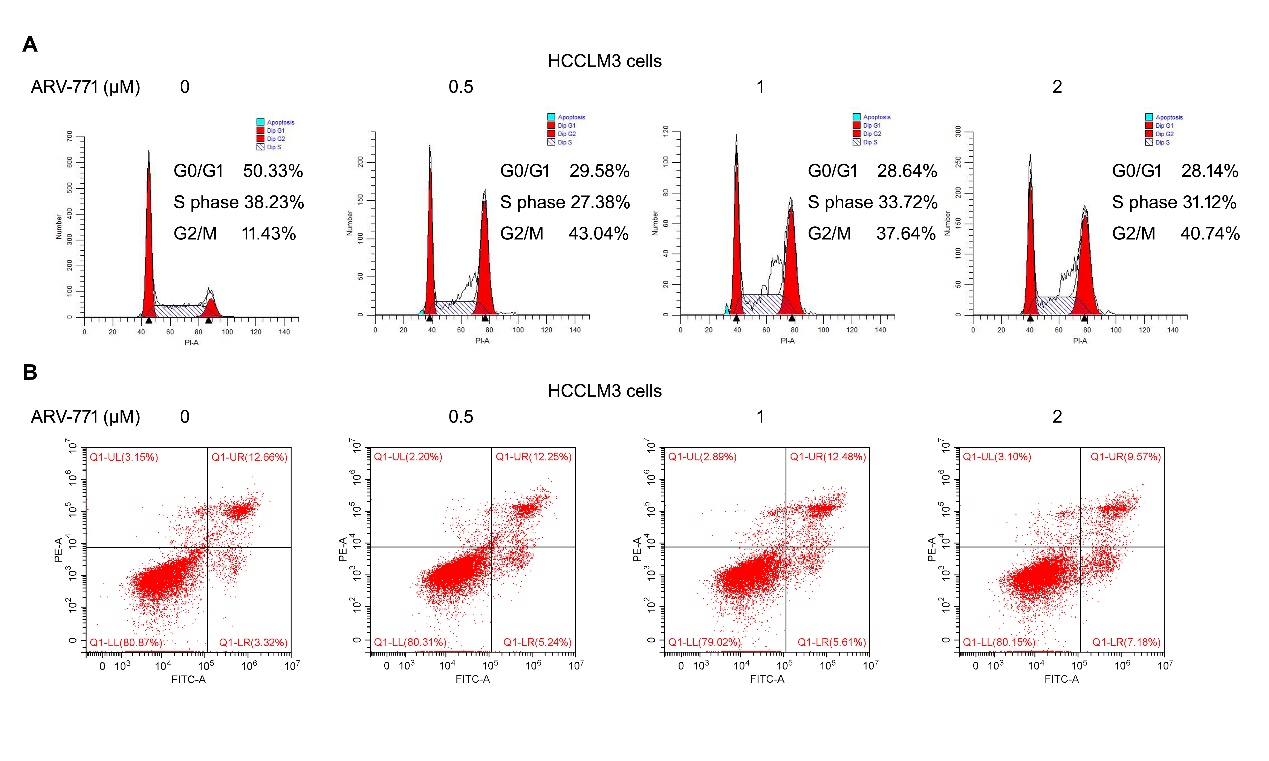


**Supplementary Figure 1. ARV-771 induces G2/M phase arrest, but fail to trigger apoptosis in HCCLM3 cells. (A)** Cell cycle assays were conducted in HCCLM3 cells exposed to ARV-771 for 24 h. **(B)** Apoptosis assays were performed in HCCLM3 cells post the treatment of ARV-771 for 24 h.


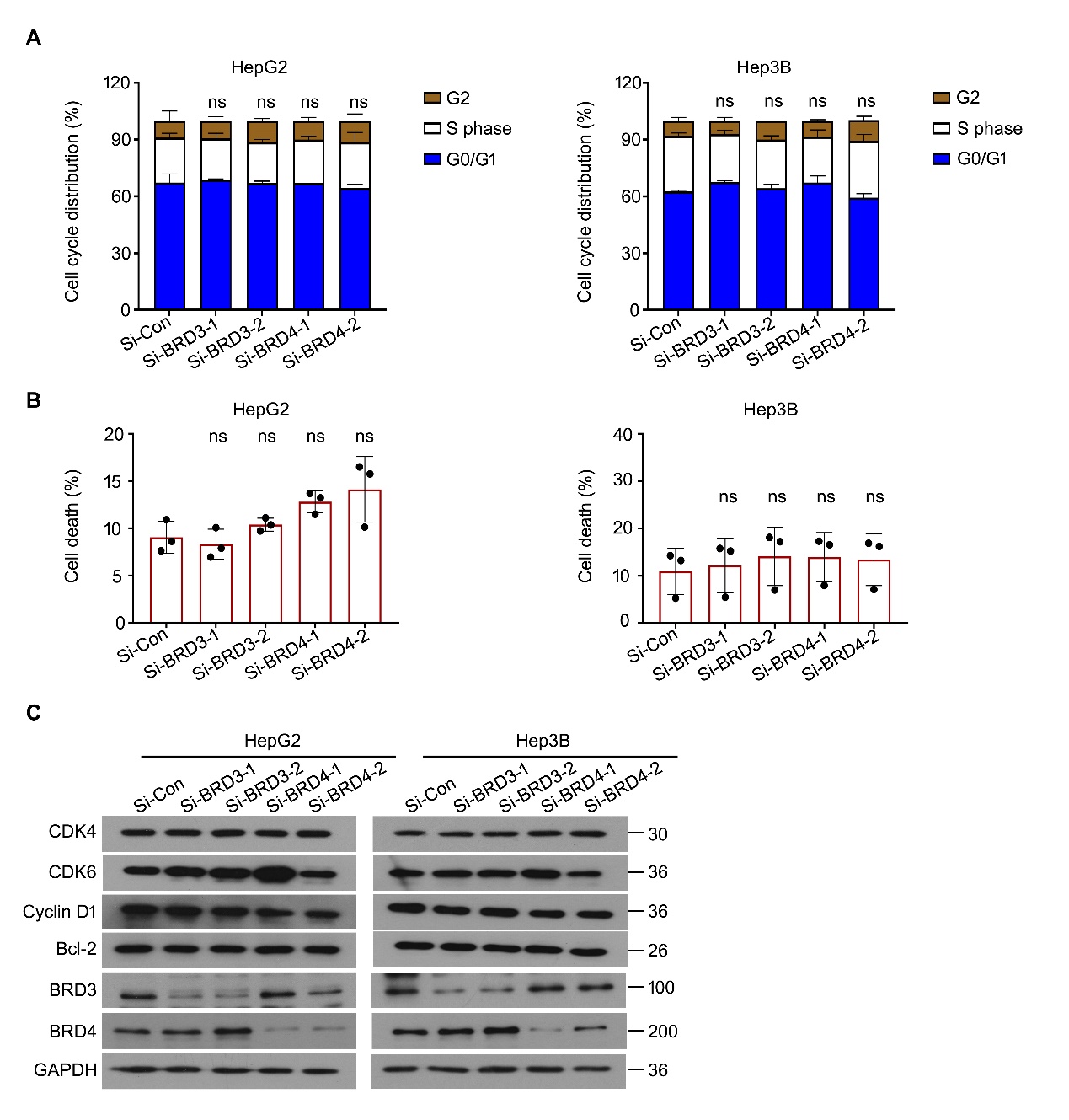


**Supplementary Figure 2. Knockdown of BRD3/4 does not induce cell cycle arrest and apoptosis in HCC cells. (A)** Cell cycle distributions, and **(B)** cell death assays were conducted in HepG2 and Hep3B cells treated with BRD3/4 siRNAs or control siRNAs for 48 h. **(C)** Western blot for CDK4/6, Cyclin D1, Bcl-2, and BRD3/4 were conducted in HepG2 and Hep3B cells treated with BRD3/4 siRNAs or control siRNAs for 48 h.


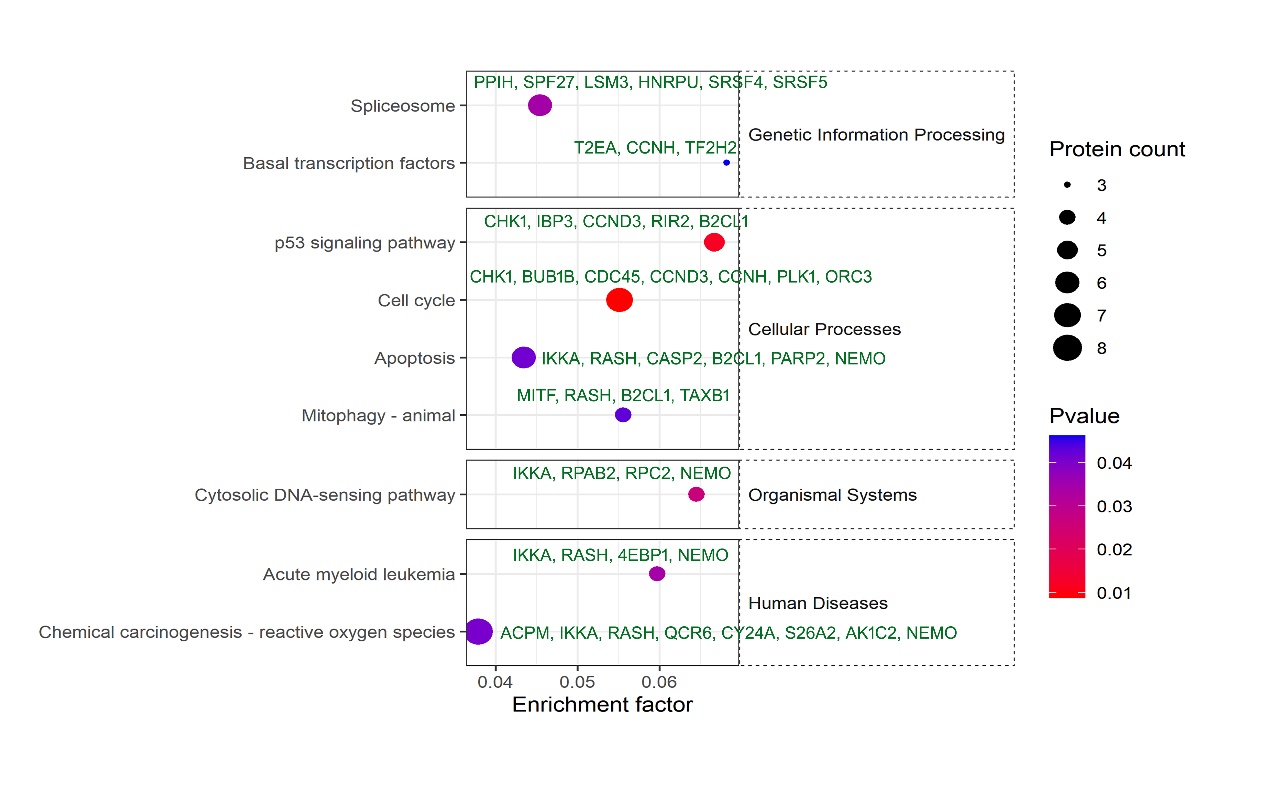


**Supplementary Figure 3. KEGG analysis post the biological mass spectrometry in HepG2 cells treated with ARV-771**.
